# Supplementary material for: Green returns to education: Does education affect pro-environmental attitudes and behaviors in China?
Source: PLoS One. 2022 Feb 3;17(2):e0263383. doi: 10.1371/journal.pone.0263383 (PMC8812898; doi:10.1371/journal.pone.0263383)
Supplement: S1 Appendix — (DOCX) [file pone.0263383.s001.docx]

**S1 Appendix. Year of implementation of compulsory schooling law across provinces**

**(DOCX)**

**Table S1. Year of implementation of compulsory schooling law across provinces**

| Year of implementation | Corresponding provinces |
| --- | --- |
| 1986 | Beijing, Hebei, Shanxi, Heilongjiang, Shanghai, Zhejiang, Jiangxi, Chongqing, Sichuan, Ningxia, Liaoning |
| 1987 | Tianjin, Jilin, Jiangsu, Anhui, Shandong, Henan, Hubei, Guangdong, Yunnan |
| 1988 | Fujian, Guizhou, Shaanxi |
| 1989 | Inner Mongolia, Qinghai |
| 1991 | Gansu |
| 1992 | Hunan, Guangxi, Hainan |
