# Supplementary material for: Green returns to education: Does education affect pro-environmental attitudes and behaviors in China?
Source: PLoS One. 2022 Feb 3;17(2):e0263383. doi: 10.1371/journal.pone.0263383 (PMC8812898; doi:10.1371/journal.pone.0263383)
Supplement: S2 Appendix — (DOCX) [file pone.0263383.s002.docx]

**S2 Appendix. Balancing test**

**(DOCX)**

**Table S2. Balancing test**

| Variables | Regression Sample | Dropped Sample | Difference |
| --- | --- | --- | --- |
|  | (1) | (2) | (3) |
| Education | 8.933  [4.607] | 8.802  [4.623] | 0.131  (0.090) |
| Male | 0.473  [0.499] | 0.480  [0.498] | -0.007  (0.009) |
| Married | 0.807  [0.395] | 0.802  [0.398] | 0.005  (0.008) |
| Income | 9.298  [4.093] | 9.299  [3.950] | -0.001  (0.079) |
| Han nationality | 0.918  [0.275] | 0.910  [0.297] | 0.008  (0.006) |
| Employed | 0.629  [0.483] | 0.637  [0.479] | -0.008  (0.010) |
| Rural | 0.488  [0.500] | 0.502  [0.498] | -0.014  (0.010) |

Note: The table reports the balancing test results. Column (1) and column (2) report the means and standard deviations (in square brackets). Column (3) reports the differences and the standard errors (in parentheses). The significance levels of 1%, 5%, and 10% are denoted by ***, **, and *, respectively.
