# Supplementary material for: Green returns to education: Does education affect pro-environmental attitudes and behaviors in China?
Source: PLoS One. 2022 Feb 3;17(2):e0263383. doi: 10.1371/journal.pone.0263383 (PMC8812898; doi:10.1371/journal.pone.0263383)
Supplement: Attachment — Submitted filename: Referee report_05.pdf [file pone.0263383.s003.pdf]

Referee report on the manuscript "Green Returns to Education: Does Education Affect Pro-Environmental Attitudes and Behaviors in China"; PLOS ONE

This paper aims to estimate the green return to education in China. Using individual data from CGSS 2010, the authors link years of schooling to pro-environmental attitudes and behaviors. To alleviate the endogeneity issue, the authors instrument education attainment with personal exposure to the implementation of the Compulsory Schooling Law in China. Based on a 2SLS model, they find that educational attainment has a moderately positive impact on individual pro-environmental attitudes and behaviors. They further provide evidence that the acquisition of environmental knowledge is one channel that explains the education effect.

Below, I outline my suggestions and comments, which hopefully will improve this paper.

1. The sample selection problem. The authors use the 2010 round of CGSS data originally containing 11,783 respondents residing in 134 cities across 31 of China's provinces. However, due to the missing values of some variables (what variables? Demographic factors or pro-environmental attitude/behaviors?), the authors drop roughly 70% of the observations without show the geographic distribution of the remaining sample, raising the concern of sample representativeness. Besides, the missing rate of outcome variables may be related to respondents' education level. For example, surveyed individuals with lower education levels may be less likely to answer questions related to pro-environmental attitudes/behaviors. If this is the case, then the analysis is derived from a selected sample and subject to external validity issues. I would suggest the authors conduct balancing checks based on all observational variables between the regression sample and the sample the author dropped. By doing this, the reader could better understand the representativeness of the regression sample.

2. The potential measurement error in pro-environmental attitude/behaviors. Well-educated people may be more likely to over-claim their pro-environmental attitudes and behaviors for keeping a good image. Then the effect of education may be overstated. The authors may include this point in the discussion/caveat section. Also, in section 4.3.2, the authors use an alternative measure of pro-environmental attitudes/behaviors to conduct a robustness check.

However, without a detailed elaboration of how to construct these new measurements, the readers are hardly convinced by this piece of evidence.

3. Specification. The current sample contains a long span of birth cohorts. Apart from the province fixed effects, I would suggest the authors include the cohort fixed effects, which captures the time-varying factors related to the pro-environmental attitudes and behaviors. For perspective, people from different cohorts may have different reporting styles, either over-claim or under-claim. They may have different preferences for cleaner air at the cost of income. They also have different techniques for learning new knowledge. Given a substantial difference among various cohorts, I feel the necessity of including the cohort fixed effects in the OLS as well as the 2SLS regressions.

4. Exclusion restrictions. The authors instrument years of schooling with personal exposure to the implementation of the Compulsory Schooling Law in China (CSL, hereafter). This IV passes the weak instrument test. However, it may not pass the requirement of exclusion restrictions. For example, given a strong relationship between educational attainment and labor outcome, treated people may have a higher expectation of their future income. People who are optimistic about their future without fear of insufficient material needs may pay more attention to environmental protection and pursuit higher quality of life. If this is the case, then CSL may affect people’s pro-environmental attitudes and behaviors through multiple channels, failing to address the endogeneity problem.

5. Heterogeneity. The authors split the sample into top quintile and bottom quintile of the income groups and check the heterogeneity by income. I am wondering whether the observations from the high-income group are mainly coming from urban areas, while those in the low-income group are from rural areas. If that is the case, then the results presented in columns (5)-(6) and (11)-(12) are explained by urban-rural status rather than income level. I would suggest the authors check the heterogeneity by income conditioning on gender and urban-rural status, and these results may provide more insight into the influence of income.

6. Typo: It should be Table 5 rather than Table 6 on page 19 paragraph 2.
