# Supplementary material for: Green returns to education: Does education affect pro-environmental attitudes and behaviors in China?
Source: PLoS One. 2022 Feb 3;17(2):e0263383. doi: 10.1371/journal.pone.0263383 (PMC8812898; doi:10.1371/journal.pone.0263383)
Supplement: Attachment — Submitted filename: PLOS report.pdf [file pone.0263383.s005.pdf]

I think the authors have done a good job revising the paper, overall. My only main remaining comment is about the mechanism.

I'm still puzzled by the fact that the positive effect is mainly driven by low-income group. If you run a simple correlation between income and environmental knowledge, I guess the correlation should be strongly positive. The authors simply rule out the "marginal utility mechanism" without any explanations. The authors could discuss the underlying story in more detail here. Additionally, I think the authors push the knowledge acquisition story somewhat too far. I do think the authors have a smoke gun on the mechanisms. I suggest the authors tone down a bit.
