# Supplementary material for: Green returns to education: Does education affect pro-environmental attitudes and behaviors in China?
Source: PLoS One. 2022 Feb 3;17(2):e0263383. doi: 10.1371/journal.pone.0263383 (PMC8812898; doi:10.1371/journal.pone.0263383)
Supplement: Attachment — Submitted filename: response to reviewers.docx [file pone.0263383.s004.docx]

We thank the editor and reviewers for providing many insightful comments. The following are our responses (comments are in italics):

**Comments from the editor:**

*Both qualified reviewers have provided constructive comments for a major revision. Please try to address their concerns as much as you can. I also have two additional suggestions. (1) If the data contain city code, you could include city fixed effects instead of province fixed effects as environmental policies and qualities are very city-specific. (2) In table 9, columns 2 and 4, you should report the coefficient of environmental knowledge variable.*

**Response:**

We have attempted to carefully address the comments from both reviewers. Please see our responses to their comments.

(1) In the revised version, we include city fixed effects in our models. We still find a positive impact of education on pro-environmental attitudes and behaviors.
(2) In the revised manuscript, we report the coefficients of the environmental knowledge variable in Columns (2) and (4) of Table 9 (mediation analysis). As expected, we find that the environmental knowledge variable has significant and positive effects on pro-environmental attitudes and behaviors.

Please see the revised manuscript for details of the revision.

**Comments from Reviewer #1:**
*1. The sample selection problem. The authors use the 2010 round of CGSS data originally containing 11,783 respondents residing in 134 cities across 31 of China’s provinces. However, due to the missing values of some variables (what variables? Demographic factors or pro-environmental attitude/behaviors?), the authors drop roughly 70% of the observations without show the geographic distribution of the remaining sample, raising the concern of sample representativeness. Besides, the missing rate of outcome variables may be related to respondents’ education level. For example, surveyed individuals with lower education levels may be less likely to answer questions related to pro-environmental attitudes/behaviors. If this is the case, then the analysis is derived from a selected sample and subject to external validity issues. I would suggest the authors conduct balancing checks based on all observational variables between the regression sample and the sample the author dropped. By doing this, the reader could better understand the representativeness of the regression sample.*

**Response:**

We thank the reviewer for pointing out the selection issue. In CGSS 2010, for questions on environmental attitudes and behaviors, only those born in February, September, November, or December are asked. Thus, our study can only use approximately one third of the sample. We conduct a balancing test based on a range of observational variables between the regression sample and the dropped sample to check whether there exists a severe sample selection issue. The results, shown in Table A2 in the appendix of the revised manuscript, suggest that the two samples are similar in observational characteristics, which gives us further confidence in the representativeness of our regression sample.

We apology for not explaining this issue clearly in the original manuscript. In the revised version, we explain how the sample is selected in more details and report the balancing test results.

Please see Section 3.1 (“Data”) and Table A2 in the revised manuscript for more details.

*2. The potential measurement error in pro-environmental attitude/behaviors. Well-educated people may be more likely to over-claim their pro-environmental attitudes and behaviors for keeping a good image. Then the effect of education may be overstated. The authors may include this point in the discussion/caveat section. Also, in section 4.3.2, the authors use an alternative measure of pro-environmental attitudes/behaviors to conduct a robustness check. However, without a detailed elaboration of how to construct these new measurements, the readers are hardly convinced by this piece of evidence.*

**Response:**

We agree with the reviewer that survey measures might suffer from measurement errors. In the conclusion section of the revised manuscript, we add a paragraph discussing limitations of our study, which include the following sentences:

“First, our study is based on survey responses, which can suffer from measurement error problems. For example, well-educated people may be more likely to over-claim their pro-environmental attitudes and behaviors for keeping a good image. If this is the case, the effect of education on actual pro-environmental attitudes and behaviors might be overstated.”

In addition, in Section 4.3.2 of the revised manuscript, we add more explanations about the rationale of the alternatives measures and the way they are constructed. We explain that factor analysis takes into account the correlations between answers to different attitudes/behaviors questions and the random error in measurement (Rummel, 1988). Specifically, we obtain an index of pro-environmental attitudes by performing a factor analysis on the three attitudes questions and an index of pro-environmental behaviors by performing a factor analysis on the six behaviors questions. The two indexes serve as alternative measures of pro-environmental attitudes and behaviors.

Please see Section 6 (“Conclusions”) and Section 4.3.2 (“Alternative pro-environmental attitudes and behaviors”) in the revised manuscript for more details.

*3. Specification. The current sample contains a long span of birth cohorts. Apart from the province fixed effects, I would suggest the authors include the cohort fixed effects, which captures the time-varying factors related to the pro-environmental attitudes and behaviors. For perspective, people from different cohorts may have different reporting styles, either over-claim or under-claim. They may have different preferences for cleaner air at the cost of income. They also have different techniques for learning new knowledge. Given a substantial difference among various cohorts, I feel the necessity of including the cohort fixed effects in the OLS as well as the 2SLS regressions.*

**Response:**

In the revised manuscript we follow the reviewer’s suggestion and control for cohort fixed effects in our model. In addition, as suggested by the editor, we also control for city fixed effects as environmental policies and qualities are city-specific. Furthermore, as suggested by another reviewer, we rescale our measures of pro-environmental attitudes and behaviors so that both measures have a maximum score of one. We still find a positive impact of education on pro-environmental attitudes and behaviors.

Please see the updated empirical results in the revised manuscript for details.

*4. Exclusion restrictions. The authors instrument years of schooling with personal exposure to the implementation of the Compulsory Schooling Law in China (CSL, hereafter). This IV passes the weak instrument test. However, it may not pass the requirement of exclusion restrictions. For example, given a strong relationship between educational attainment and labor outcome, treated people may have a higher expectation of their future income. People who are optimistic about their future without fear of insufficient material needs may pay more attention to environmental protection and pursuit higher quality of life. If this is the case, then CSL may affect people’s pro-environmental attitudes and behaviors through multiple channels, failing to address the endogeneity problem.*

**Response:**

(1) We understand the concern of the reviewer. However, we see the example proposed by the reviewer as an alternative channel underlying the relation between education and pro-environment attitudes and behaviors rather than a threat to identification. If increased optimism is a result of more years of schooling (due to more exposure to CSL), and, optimism can increase the intensity of pro-environmental attitudes and behaviors, then we can still argue that education has a causal impact on pro-environmental attitudes and behaviors (by increasing optimism). We acknowledge that the knowledge channel we focus on in this study might not be the only channel. In the last paragraph of the conclusion section, we add the following:

“In addition, partly due to data availability, we focus on the knowledge acquisition channel. There might exist other channels underlying the relationship between education and pro-environmental attitudes and behaviors. For example, given the strong relationship between educational attainment and labor market outcomes, people with a higher educational attainment might be more optimistic about their future income, and thus have less concern about insufficient material needs when following a pro-environmental lifestyle.”

(2) In addition, we agree with the reviewer that it is difficult to empirically prove that the exclusion restrictions are completely satisfied. In fact, in general the exclusion restrictions in IV models are not testable. In the last paragraph of the conclusion section, we add the following discussion:

“Moreover, as in general the exclusion restrictions in IV models are not testable (Angrist and Krueger, 2001), other research methods (e.g., lab and field experiments) can be useful to validate our findings.”

Please see the last paragraph of Section 6 (“Conclusions”) in the revised manuscript for more details.

*5. Heterogeneity. The authors split the sample into top quintile and bottom quintile of the income groups and check the heterogeneity by income. I am wondering whether the observations from the high-income group are mainly coming from urban areas, while those in the low-income group are from rural areas. If that is the case, then the results presented in columns (5)-(6) and (11)-(12) are explained by urban-rural status rather than income level. I would suggest the authors check the heterogeneity by income conditioning on gender or urban-rural status, and these results may provide more insight into the influence of income.*

**Response:**

(1) In the revised manuscript, we explain that after splitting the sample based on a given individual characteristic, within each sub-sample we regress pro-environmental attitudes and behaviors on education, controlling for other observable characteristics.
(2) We also check the distribution of rural and urban residents in high-come and low-income groups. Although urban residents tend to have higher income, there are still a certain amount of rural population in the high-income group. For example, with respect to the regressions of pro-environmental attitudes (Columns 5 and 6 in Table 5 of the revised manuscript), in the high-income group there are 458 urban residents and 260 rural residents and in the low-income group there are 187 urban residents and 469 rural residents.
(3) In the manuscript, the high-income group includes those in the top quintile of the income distribution and the low-income group includes those in the bottom quintile. We have also checked the results by splitting the sample based on median income, so that the two groups have a more balanced rural-urban distribution. We find a similar result. The results are shown below for the reviewer’s information:

Heterogeneity analysis based on median income.

|  | Pro-environmental attitudes | | Pro-environmental behaviors | |
| --- | --- | --- | --- | --- |
|  | High income | Low income | High income | Low income |
|  | (1) | (2) | (3) | (4) |
| Education | 0.007 | 0.016* | 0.001 | 0.019** |
|  | (0.007) | (0.009) | (0.007) | (0.008) |
| Controls | Yes | Yes | Yes | Yes |
| City FE | Yes | Yes | Yes | Yes |
| Observations | 1,716 | 1,740 | 1,820 | 1,841 |
| **First-stage estimation** |  |  |  |  |
| CSL | 2.129*** | 3.438*** | 2.358*** | 3.767*** |
|  | (0.332) | (0.493) | (0.348) | (0.478) |
| F-statistics | 40.94 | 48.58 | 45.86 | 62.03 |

We do not include the above table in the manuscript for brevity, only leaving a footnote explaining this point (footnote 3).

Please see Section 4.2. (“Heterogeneity analysis”) in the revised manuscript for more details.

*6. Typo: It should be Table 5 rather than Table 6 on page 19 paragraph 2.*

**Response:**

We correct this typo in the revised manuscript.

**Reference**

Angrist, J. D., & Krueger, A. B. (2001). Instrumental variables and the search for identification: From supply and demand to natural experiments. *Journal of Economic Perspectives*, *15*(4), 69-85.

Rummel, R. J. (1988). *Applied Factor Analysis*. Northwestern University Press.

**Comments from Reviewer #2:** *1. Contribution and the introduction part
There is abundant literature documenting the relationship between education attainment and social attitudes or behaviors related to environmental protection. The marginal contribution of this article is to deal with the endogeneity of education in this context. How can we compare the findings of this paper with previous literature? Are the estimates of education attainment different with studies which fail to deal with endogeneity problem or use data from other countries? It is important to understand how different or unique the findings are when we focus on China. A more comprehensive literature review could be helpful.
Moreover, is there any theory to predict the relationship between education attainment and pro-social behaviors? Can education attainment negatively affect pro-environmental behaviors or they have a non-linear relationship? The authors could refer to a broader scope of literature and motivate the research question more carefully.*

**Response:**

We thank the reviewer for pushing us on this important issue. In the revised manuscript, we devote more effort and space to discuss the literature and our contributions.

(1) In the second section of the original manuscript, we only explain the background of CSL in China. In the revised version, we change the section title to “Literature review and institutional background” and begin with a comprehensive discussion of the literature.
(2) In the revised Section 2, we first link our study to the broad literature on non-pecuniary and social returns to education (McMahon, 2004; Lange and Topel, 2006; Oreopoulos et al., 2011).
(3) Then, we discuss the theories behind the potential relationship between education and pro-environment attitudes and behaviors. We mention that formal schooling is the primary way individuals acquire knowledge and skills (Chankrajang and Muttarak, 2017). In addition, people with higher level of formal education have access to more sources and types of information (Wen et al., 2011). Moreover, we introduce the hierarchy of needs theory (Maslow, 1970; Van Liere and Dunlap, 1980), based on which we argue that education might help individuals meet their basic needs, and thereby enabling them to adopt a pro-environmental lifestyle to pursue a higher level of demand (Inglehart, 1995; Ponce et al., 2019). In another word, education might increase the marginal utility of pro-environmental behaviors relative to the marginal utility of money.
(4) To take a balanced view, we also argue that in some cases education might also be detrimental to the development of pro-environmental attitudes and behaviors. In East Asia and, in particular, China, schooling education is examination-oriented and emphasizes competition (Zhao, 2015; Kajanus, 2019). Individuals who become highly competitive might focus on self-enhancement values such as power and achievement (Lu et al., 2013) and are therefore less caring for the environment.
(5) We then further motivate our study by arguing that a causal analysis can provide important public policy implications and China offers an interesting context to study this topic. China is facing many severe environmental problems (Xu et al., 2012; Zhang and Wu, 2012; Yu et al., 2019). As the second largest economy in the world, China’ environmental problems can have global impacts. In addition, China has its own unique culture and ideology, which are deeply embedded in its education system. Therefore, studying the Chinese context helps to evaluate the external validity of findings in prior studies.
(6) We also revise the introduction section to incorporate the related theories and highlight our contributions.

Please see Section 1 (“Introduction”) and Section 2 (“Literature review and institutional background”) in the revised manuscript for more details.

*2. Birth cohort fixed effects
The age range of respondents in the sample is large. Respondents from different cohorts vary greatly in their social attitudes. By including the age variable may not be enough to control unobserved heterogeneity across birth cohorts. As individuals exposed to CSL are from younger sample, I’m a bit concerned that the major finding that more education attainment promotes pro-environmental attitudes is actually driven by birth cohort variations. I suggest that the authors control cohort fixed effects in the regression, for example, constructing dummies for every 10-year age interval.*

**Response:**

In the revised manuscript, we follow the reviewer’s suggestion and control for cohort fixed effects in our model. In addition, as suggested by the editor, we also control for city fixed effects as environmental policies and qualities are city-specific. We still find a positive impact of education on pro-environmental attitudes and behaviors.

Please see the updated empirical results in the revised manuscript for details.

*3. Interpretation of OLS and IV estimates
According to Table 3 and Table 4, we know that if we ignore the endogeneity problem of education OLS will overestimate the effect on pro-environmental attitudes but underestimate that on pro-environmental behaviors. How can we interpret this? Please carefully discuss how OLS estimates and IV estimates are different and why.*

**Response:**

Under the model specification in the revised manuscript, we find that the IV estimates are larger than the corresponding OLS estimates for regressions of pro-environmental attitudes and behaviors. We attempt to provide some explanations. Some studies suggest that measurement errors in schooling can lead to downward bias in the OLS estimate of the effect of schooling (Griliches, 1977; Card, 2001). In addition, some unobservable traits might be positively related to educational attainment and negatively related to pro-environmental attitudes and behaviors, making the OLS estimates underestimate the positive impacts of education. For example, an orientation towards personal achievement might be positively related to educational attainment in China, as the East Asian education model centers around competition and performance goals (Zhu & Leung, 2011). People that are obsessed with personal achievement might care less for the environment.

Please see Section 4.1. (“Benchmark results”) in the revised manuscript for more details.

*4. Alternative mechanism of the findings
To interpret the major findings, the authors test the environmental knowledge mechanism among others. The mediation analysis shows that more years of education improves environmental knowledge, hence promoting pro-environmental attitudes and behaviors. But an alternative mechanism could be that individuals with more education attainment have higher incomes, so the marginal utility of pro-social behaviors relative to marginal utility of money is larger for them than that for the poor. As a matter of fact, the authors already did a heterogeneity analysis by splitting the high-income and the low-income. From Table 5 we can know that the positive effect of education attainment is mainly driven by the low-income group. This implies that it is environmental knowledge which plays a role, not marginal utility of alleviating environmental problems. The authors could discuss the mechanisms in more detail here.*

**Response:**

(1) We thank the reviewer for pointing out this issue. In the revised manuscript, we introduce the marginal utility mechanism in the second paragraph of Section 2 (“Literature review and institutional background”) when we discuss the potential mechanism.
(2) In addition, we discuss the implications of the results of the heterogeneity analysis by splitting the high-income and the low-income. We add the following sentences in the last paragraph of Section 4.2 (“Heterogeneity analysis”):

“The estimate of schooling years is positive and significant for people in low-income groups, but insignificant for those with high income. This finding seems to contradict the argument that the positive impact of education on pro-environmental behaviors is because education increase peoples’ income and people with higher income have a higher marginal utility of pro-social behaviors. In later section of this paper we will explore an alternative channel, which is the knowledge acquisition channel.”

Please see Section 2 (“Literature review and institutional background”) and Section 4.2 (“Heterogeneity analysis”) in the revised manuscript for more details.

*5. Scale of measures
It is not very clear why and how the authors construct the attitudes questions into a variable ranging from 1 to 15 and the behaviors questions into a variable ranging from 1 to 18. Why not construct two variables of the same range, or just use the original survey questions as dependent variables? Are the findings robust against methods of constructing dependent variables?
In addition, I suggest that maximum and minimum of all variables could also be reported in Table 2.*

**Response:**

(1) In the revised manuscript, we provide more explanations about the construction of the dependent variables in Section 3.2.1 (“Pro-environmental attitudes and behaviors”).

“Following the previous literature (e.g., Meyer, 2015), we aggregate the three attitudes questions into one variable. For each of the three attitude questions, respondents are asked to provide answers on a five-point scale from “Extremely unwilling to” (i) to “Extremely willing to” (v), with a higher value indicating a stronger pro-environmental attitude. By summing answers to the three questions together, we obtain a variable with a maximum score of 15. We divide this variable by 15 to construct the variable *Pro-environmental attitudes*, which has a maximum score of one. Similarly, we aggregate the six behavior questions into one variable. For each of the six behavior questions, respondents provide answers on a three-point scale, with a higher value indicating a higher intensity of pro-environmental behaviors. We construct the variable *Pro-environmental behaviors* by summing answers to the six behavior questions and divide the result by 18, which makes the variable has a maximum value of one. All else being equal, individuals who have higher values of pro-environmental attitudes and behaviors are likely to think and behave in a more environmentally friendly manner.”

In the revised manuscript, we use the rescaled measures of pro-environmental attitudes and behaviors (both measures have a maximum value of one). In addition, in the robustness section we construct measures of pro-environmental attitudes and behaviors using factor analysis. We find that the results are robust to different methods of constructing dependent variables.

(2) In the revised manuscript we report the maximum and minimum of variables in Table 2.

Please see Section 3.2.1 (“Pro-environmental attitudes and behaviors”), Section 4.3.2. (“Alternative pro-environmental attitudes and behaviors”), and Table 2 in the revised manuscript for details.

*6. Missing sample
The sample used in this analysis is 3,661 observations, around 30% of the full CGSS 2010 sample. Are the respondents randomly chosen to answer the pro-environmental questions? More explanation how the sample is constructed could be included.*

**Response:**

In CGSS 2010, for questions on environmental attitudes and behaviors, only those born in February, September, November, or December are asked. Thus, our study can only use approximately one third of the sample. We conduct a balancing test based on a range of observational variables between the regression sample and the dropped sample to check whether there exists a severe sample selection issue. The results, shown in Table A2 in the appendix of the revised manuscript, suggest that the two samples are similar in observational characteristics, which gives us further confidence in the representativeness of our regression sample.

In the original manuscript we did not explain the sample selection issue clearly. In the revised version, we explain how the sample is selected in more details and report the balancing test results.

Please see Section 3.1 (“Data”) and Table A2 in the revised manuscript for more details.

**Reference**

Card, D. (2001). Estimating the return to schooling: Progress on some persistent econometric problems. *Econometrica*, *69*(5), 1127-1160.

Chankrajang, T., & Muttarak, R. (2017). Green returns to education: Does schooling contribute to pro-environmental behaviours? Evidence from Thailand. *Ecological Economics*, *131*, 434-448.

Griliches, Z. (1977). Estimating the returns to schooling: Some econometric problems. *Econometrica: Journal of the Econometric Society*, 1-22.

Inglehart, R. (1995). Public support for environmental protection: Objective problems and subjective values in 43 societies. *PS: Political Science & Politics*, *28*(1), 57-72.

Kajanus, A. (2019). Mutualistic vs. zero‐sum modes of competition-a comparative study of children's competitive motivations and behaviours in China. *Social Anthropology*, *27*(1), 67-83.

Lange, F., & Topel, R. (2006). The social value of education and human capital. *Handbook of Education Economics*, *1*, 459-509.

Liere, K. D. V., & Dunlap, R. E. (1980). The social bases of environmental concern: A review of hypotheses, explanations and empirical evidence. *Public opinion quarterly*, *44*(2), 181-197.

Lu, S., Au, W. T., Jiang, F., Xie, X., & Yam, P. (2013). Cooperativeness and competitiveness as two distinct constructs: Validating the cooperative and competitive personality scale in a social dilemma context. *International Journal of Psychology*, *48*(6), 1135-1147.

Maslow, A. H. (1970). New introduction: Religions, values, and peak-experiences. *Journal of Transpersonal Psychology*, *2*(2), 83-90.

McMahon, W. W. (2004). The social and external benefits of education. *International Handbook on the Economics of Education*, *211*, 259.

Meyer, A. (2015). Does education increase pro-environmental behavior? Evidence from Europe. *Ecological Economics*, *116*, 108-121.

Oreopoulos, P., & Salvanes, K.G.. (2011). Priceless: The nonpecuniary benefits of schooling. *Journal of Economic Perspectives*, *25*(1), 159-184.

Ponce, P., Alvarado, R., Ponce, K., Alvarado, R., Granda, D., & Yaguana, K. (2019). Green returns of labor income and human capital: Empirical evidence of the environmental behavior of households in developing countries. *Ecological Economics*, *160*, 105-113.

Wen, L.M., Rissel, C., Baur, L.A., Lee, E., Simpson, J.M. (2011). Who is NOT likely to access the internet for health information? Findings from first-time mothers in southwest Sydney, Australia. *International Journal of Medical Informatics*, *80*, 406-411.

Xu, P., Zeng, Y., Fong, Q., Lone, T., & Liu, Y. (2012). Chinese consumers’ willingness to pay for green- and eco-labeled seafood. *Food Control*, *28*(1), 74-82.

Yu, X., Lei, X., & Wang, M. (2019). Temperature effects on mortality and household adaptation: Evidence from China. *Journal of Environmental Economics and Management*, *96*, 195-212.

Zhang, L., & Wu, Y. (2012). Market segmentation and willingness to pay for green electricity among urban residents in China: The case of Jiangsu Province. *Energy Policy*, *51*, 514-523.

Zhao, X. (2015). Competition and compassion in Chinese secondary education. New York, NY, USA: Palgrave Macmillan.

Zhu, Y., & Leung, F. K. (2011). Motivation and achievement: Is there an East Asian model?. *International Journal of Science and Mathematics Education*, *9*(5), 1189-1212.
