# Supplementary material for: Green returns to education: Does education affect pro-environmental attitudes and behaviors in China?
Source: PLoS One. 2022 Feb 3;17(2):e0263383. doi: 10.1371/journal.pone.0263383 (PMC8812898; doi:10.1371/journal.pone.0263383)
Supplement: Attachment — Submitted filename: response to reviewers.docx [file pone.0263383.s006.docx]

We thank the editor and reviewers for providing many insightful comments. The following are our responses (comments are in italics):

**Comments from the editor:**

*1. After careful consideration, we feel that it has merit but does not fully meet PLOS ONE’s publication criteria as it currently stands. Both the reviewers still raised some minor concerns. Therefore, we invite you to submit a revised version of the manuscript that addresses the points raised during the review process.*

**Response:** Thanks for giving us the opportunity to further polish the paper. In the revised version we have attempted to carefully address the remaining concerns raised by both reviewers. Please see our response letter for details.

*2. Journal Requirements:*

*Please review your reference list to ensure that it is complete and correct. If you have cited papers that have been retracted, please include the rationale for doing so in the manuscript text, or remove these references and replace them with relevant current references. Any changes to the reference list should be mentioned in the rebuttal letter that accompanies your revised manuscript. If you need to cite a retracted article, indicate the article’s retracted status in the References list and also include a citation and full reference for the retraction notice.*

**Response:** In the revised version we reviewed our reference list to ensure its completeness and correctness. Please see the references section for more details.

**Comments from Reviewer #1:**
*1. I think the authors have done a good job revising the paper, overall. My only main remaining comment is about the mechanism.*

*I’m still puzzled by the fact that the positive effect is mainly driven by low-income group. If you run a simple correlation between income and environmental knowledge, I guess the correlation should be strongly positive. The authors simply rule out the “marginal utility mechanism” without any explanations. The authors could discuss the underlying story in more detail here. Additionally, I think the authors push the knowledge acquisition story somewhat too far. I do think the authors have a smoke gun on the mechanisms. I suggest the authors tone down a bit.*

**Response:**

In the revised version, we provided more discussions on the finding that the positive effect is mainly driven by low-income group.

First, it should be noted that in the heterogeneity analysis, we are comparing the effects of education between different income groups. We are not evaluating the effect of income itself on environmental attitudes and behaviors. Therefore, whether income is positively related to environmental knowledge it not the focus of this analysis. We agree, however, that the fact that the positive effect is mainly driven by low-income group does not rule out the “marginal utility mechanism”. Therefore, in this version we deleted the statement “This finding seems to contradict the argument that the positive impact of education on pro-environmental behaviors is because education increase peoples’ income and people with higher income have a higher marginal utility of pro-social behaviors”.

Second, in the revised version we provided more discussions on the findings of the heterogeneity analysis. Prior studies show that people’s demand for public goods such as clean environment will increase with income (e.g., Kotchen et al., 2013). Accordingly, high income people might already have a high level of environmental awareness, so that the effect of additional schooling years become negligible. Put it in another word, our finding suggests that income can substitute for the effect of education.

Finally, in this study what we find is that knowledge acquisition is an important channel underlying the relationship between education and pro-environmental attitudes and behaviors. However, we do not rule out the possibility that there exists other (coexisting) channels. Following the reviewer’s suggestion, we tone down a bit in the revised version. In particular, we acknowledged this issue and discussed the limitation of the paper in the conclusion section.

Please see the last paragraph of Section 4.2 (“Heterogeneity analysis”) in the revised manuscript for more details.

**Reference**

Kotchen, M. J., Boyle, K. J., & Leiserowitz, A. A. (2013). Willingness-to-pay and policy-instrument choice for climate-change policy in the United States. Energy Policy, 55, 617-625.

**Comments from Reviewer #2:** *The current manuscript addresses my comments well by providing a more comprehensive literature review, including richer background information and conducting more rigorous regression analysis. However, there are several comments that the authors can take into further account before making it to a publishable version.*

*1. Page 8, line 6: “…but also because China’s unique culture and institution help validate...”
Page 11, line 18: “In addition, China has its own unique culture and ideology, …”
It is not very clear which part of China’s unique cultural or institutional features would help validate the relationship between education and environmental attitudes. The authors should avoid too general arguments without further elaboration in a scientific research paper. Instead, the authors could briefly discuss China’s environmental problems as a consequence of fast industrial growth as part of motivation. That is a unique feature of the Chinese context.*

**Response:**

Thanks for the comments. We agree that we should avoid too general arguments without further elaboration. In the revised manuscript, we dropped the sentences mentioned by the reviewer.

Following the reviewer’s suggestion, we add the following sentences in the introduction section to briefly discuss China’s environmental problems as a consequence of fast industrial growth:

“As the cost of fast industrial growth and rapid urbanization, China has suffered a series of environmental problems (Zheng and Kahn, 2013). As the largest developing country in the world, the environmental problems of China have strong global impacts. Environmental attitudes and behaviors of Chinese residents play an important role in affecting the country’s future environmental scenarios.”

Please see the sixth paragraph in Section 1 for more details.

2. *Page 11, line 5: “…in particular, China, schooling education is examination-oriented and emphasizes competition… are therefore less caring for the environment”
Page 23, line 8: “people that are obsessed with personal achievement might care less for the environment.”
There is no solid evidence to support the negative association between education and pro-environmental attitudes. The authors should elaborate very carefully how willingness to compete conflicts with pro-environmental attitudes, and why this is unique in China. As a matter of fact, I think there is no need to raise this point. The downward bias of OLS estimates might be driven by measurement error. I would prefer not to mention this channel.*

**Response:**

We agree with the reviewer that it is difficult to image a negative association between education and pro-environmental attitudes. Following the reviewer’s suggestion, in the revised version we dropped the discussion on the channel mentioned above. Instead, we mentioned that the downward bias of OLS estimates might be driven by measurement error in survey response.

Please see the last paragraph of Section 4.1 (“Benchmark results”) in the revised manuscript for details.

*3. Page 14: why are only those born in February, September, November or December asked about environmental attitudes and behaviors in CGSS? It is difficult to understand.*

**Response:**

To shorten questionnaire length and improve survey compliance, some modules in CGSS 2010 are assigned to a subset of respondents. For example, for questions on environmental attitudes and behaviors, only those born in February, September, November, or December are asked. Those born in other months are assigned some other modules.

In the revised version we provided more explanations on this point. Please see the second paragraph in Section 3.1 (“Data”) for more details.

*4. Typos
page 7, line 6: “While there are large number of papers…” should be “there are a large number of …”
page 10, line 13: “… skills accumulated through education make individuals be familiar with…”, delete “be”
page 10, line 15: “… people with higher level of formal education…”, should be “with a higher level”*

**Response:**

We thank the reviewer for pointing out those typos. We have corrected all the typos mentioned by the reviewer.

**Reference**

Zheng, S., & Kahn, M. E. (2013). Understanding China's urban pollution dynamics. Journal of Economic Literature, 51(3), 731-72.
